# Center Volumes Correlate with Likelihood of Stent Implantation in German Coronary Angiography

**DOI:** 10.1155/2023/3723657

**Published:** 2023-11-09

**Authors:** Vera Oettinger, Philip Hehn, Christoph Bode, Manfred Zehender, Constantin von zur Mühlen, Dirk Westermann, Peter Stachon, Klaus Kaier

**Affiliations:** ^1^Department of Cardiology and Angiology, University Heart Center, Medical Center–University of Freiburg, Faculty of Medicine, University of Freiburg, Freiburg, Germany; ^2^Center for Big Data Analysis in Cardiology (CeBAC), Department of Cardiology and Angiology, University Heart Center, Medical Center–University of Freiburg, Faculty of Medicine, University of Freiburg, Freiburg, Germany; ^3^Institute of Medical Biometry and Statistics, Faculty of Medicine, Medical Center–University of Freiburg, Freiburg, Germany

## Abstract

**Aims:**

Literature on percutaneous coronary intervention (PCI) stated an inverse relationship between hospital volume and mortality, but the effects on other characteristics are unclear.

**Methods:**

Using German national records, all coronary angiographies with coronary artery disease in 2017 were identified. We applied risk-adjustment to account for differences in population characteristics.

**Results:**

Of overall 528,188 patients, 55.22% received at least one stent, with on average 1.01 stents implanted in all patients. Based on those patients who received at least one stent, this corresponds to an average number of 1.82 stents. In-hospital mortality across all patients was 2.93%, length of hospital stay was 6.46 days, and mean reimbursement was €5,531. There were comparatively more emergency admissions in low volume centers and more complex cases (3-vessel disease, left main stenosis, and in-stent stenosis) in high volume centers. In multivariable regression analysis, volume and likelihood of stent implantation (*p*=0.003) as well as number of stents (*p*=0.020) were positively correlated. No relationship was seen for in-hospital mortality (*p*=0.105), length of stay (*p*=0.201), and reimbursement (*p*=0.108). Nonlinear influence of volume suggests a ceiling effect: In hospitals with ≤100 interventions, likelihood and number of implanted stents are lowest (∼34% and 0.6). After that, both rise steadily until a volume of 500 interventions. Finally, both remain stable in the categories of over 500 interventions (∼60% and 1.1).

**Conclusion:**

In PCI, lower volume centers contribute to emergency care. Higher volume centers treat more complex cases and show a higher likelihood of stent implantations, with a stable safety.

## 1. Introduction

The impact of the intervention volume on outcomes of coronary angiography and percutaneous coronary intervention (PCI) has been a prominent subject of medical interest for a long time. A considerable body of previous literature investigating the volume-outcome relationship in PCI in various countries exists, approaching the issue from the center and operator perspective. Data sources include registries of sizes up to national cohorts as well as administrative data sources. Recent reviews describe that operator volumes and hospital volumes, respectively, influence rates of mortality as well as major adverse cardiac events in PCI [1–3], and the guidelines for the United States [4], the UK [5], and Europe [6] contain volume requirements. On the one hand, high volume centers might combine more extensive experience and advanced technology leading to optimal procedural outcomes, which is of particular importance in complex cases such as 3-vessel or left main disease. On the other hand, low volume centers may have a relevant role in emergency care, particularly in regions without easy access to a cardiac catheterization laboratory. Even with possibly less expertise or technology, low volume centers could ensure timely treatment [2]. The question however remains of interest since procedural numbers cannot be relied on to remain constant or increasing [7, 8], with any decrease making it difficult for some of the existing centers and operators to meet the minimum thresholds. The data provided in this study present tools to help navigating this conflict.

Since further progress in coronary angiography and PCI was achieved during the last decade continuing high-quality, methodologically sound [9] scrutiny of the volume-outcome effect in the real-world clinical practice of PCI is warranted. We investigate the impact of center volume on in-hospital mortality, likelihood of stent implantation, number of stents, length of hospital stay, and reimbursement in a nationwide German cohort. In contrast to the previous literature, a documented coronary artery disease (CAD) and coronary angiography serve as inclusion criteria, so that stent implantation can be used as an endpoint rather than an inclusion criterion.

## 2. Methods

### 2.1. Data

Since 2005, all hospitalization data in Germany is obtainable for scientific use via the diagnosis-related groups (DRG) statistics of the Research Data Center of the Federal Bureau of Statistics (DESTATIS). This representative data are a valuable source for analyzing the in-hospital treatment of patients [10, 11].

This source represents a virtually complete database of all hospitalizations in Germany that are reimbursed within the DRG system. We extracted the data on all PCIs performed in Germany in the year 2017 for our analysis. In detail, we included all patients who were hospitalized in 2017 with a documented coronary artery disease (ICD-10 code I25.11, I25.12, I25.13, and I25.14 as main or secondary diagnosis) who also underwent a coronary angiography (German Operation and Procedure Classification/OPS code 1–275).

Our study did not involve immediate investigator access to individual patient data, only access to summary results provided by DESTATIS. Therefore, it was determined that approval by an ethics committee and informed consent were not required, in accordance with the German law. All summarized results were anonymized by DESTATIS. This means that all information that allows the drawing of conclusions regarding an individual patient or a specific hospital is censored by DESTATIS to ensure data protection. In particular, the use of the anonymous, persistent “institute indicator of hospitals” is severely restricted in order not to publish any information that can be directly assigned to a single hospital [10, 12].

Primary outcome was the in-hospital mortality. Secondary outcomes include the likelihood of stent implantation per patient, i.e., the probability at least one stent being implanted, the number of implanted stents (summed according to OPS codes 8-837.m0 to 8-837.ma), length of hospital stay, and total reimbursement. Procedure volume per center was calculated on the basis of an anonymous, persistent “institute indicator of hospitals,” provided by DESTATIS.

### 2.2. Multivariable Regression

In order to determine the impact of center volumes on the various endpoints, multivariable logistic (in-hospital mortality and likelihood of stent implantation) or linear (number of implanted stents, length of hospital stay, or total reimbursement) regression analyses were carried out. A total of 13 baseline patient characteristics were included as potential confounders (all covariates listed in Table 1). The question of how to account for the correlation of error terms of patients treated in the same hospital was discussed previously [13–15]. As recommended in a previous study that also used data from the German DRG-statistic [15], we used cluster-robust standard errors to account for this dependency.

In the first step, procedure volumes per center were included as continuous covariates in the abovementioned multiple regression models. In the second step, the nonlinear impact of center volumes was explored using categorization procedure volumes in steps of 100 procedures, from ≤100 to >1000. In order to visualize the nonlinear impact of center volumes, predicted probabilities have been calculated using marginal standardization (prediction at the means, see Table 1 for mean values of all confounders) [16]. The visualization of these risk-adjusted rates or means together with their 95% confidence intervals constitutes the main analytical approach in this paper. All analyses were carried out using Stata 16.0 (StataCorp, College Station, Texas, USA).

## 3. Results

We analyzed 528,188 procedures conducted in 828 German centers in 2017. Mean patient age was 69.79 years and mean EuroSCORE was 8.43. Only 29.27% of the patients were women. Regarding the coronary artery disease, most patients were classified 3-vessel CAD (45.39%). Furthermore, non-ST-segment elevation myocardial infarction (NSTEMI) was present in 20.14% of the patients, while 11.20% of the patients had an ST-segment elevation myocardial infarction (STEMI). 50.12% of hospitalizations in which coronary angiography took place were coded as emergency admissions (Table 1).

**Figure 1:**
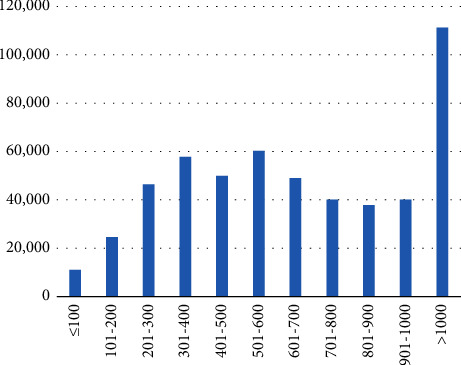
Number of patients undergoing coronary angiography in low and high volume centers in 2017.

As shown in Figure 1, 2.09% (*N* = 11,049) of the patients underwent coronary angiography in one of the 281 low volume centers with less than 100 conducted PCIs. In contrast, 21.06% (*N* = 111,234) underwent coronary angiography in one of the 44 centers from the highest volume category with more than 1000 conducted PCIs.

**Figure 2:**
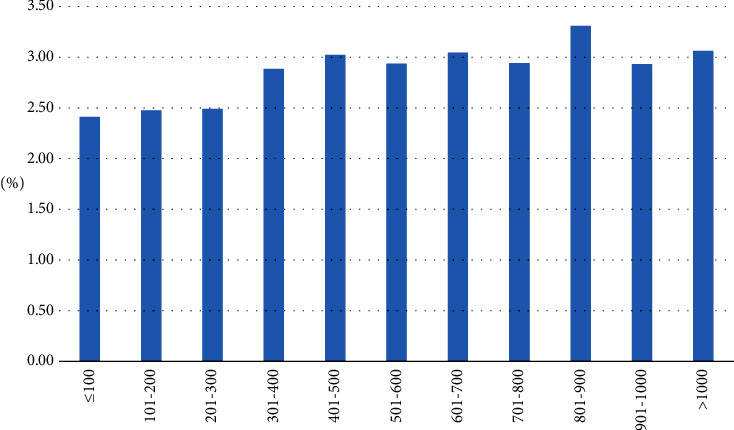
Unadjusted in-hospital mortality of patients undergoing coronary angiography in low and high volume centers in 2017.

As shown in Table 2, 55.22% of the patients undergoing coronary angiography with a diagnosis of a coronary artery disease received at least one stent. The mean number of stents implanted in all patients suffering from any coronary heart disease was 1.01. Based on those patients who received at least one stent, this corresponds to an average number of 1.82 stents per patient. In-hospital mortality across all patients was 2.93%, ranging between 2.41 and 3.30% (Figure 2). The mean length of stay was 6.46 days, and the average reimbursement was €5,531.

As seen in the multivariable regression analysis (Supplementary Data S1), significantly associated with higher in-hospital mortality were STEMI (risk adjusted OR = 8.47 [95% CI 7.93; 9.04], *p* < 0.001), NSTEMI (OR = 2.60 [2.44; 2.77], *p* < 0.001), left main stenosis (OR = 1.67 [1.57; 1.77], *p* < 0.001), and emergency admission (OR = 1.43 [1.33; 1.54], *p* < 0.001).

### 3.1. Center Volume and Patient Population

In the low volume centers, comparatively more acute cases with NSTEMI or those marked as emergency as well as more symptomatic patients with unstable angina pectoris were treated.

However, high volume centers treated patients with more severe coronary artery disease: In comparison of the lowest to highest categories, the share of 1-vessel CAD drops from 31.95% to 24.22%, whereas the share of 3-vessel CAD increases from 39.52% to 49.08%. In parallel, the rates of left main stenosis rose from 5.33% to 9.18% and in-stent stenosis from 1.70% to 6.42% (Table 1).

### 3.2. Center Volume, Stent Rate, and Outcomes

In multivariable regression analysis, center volume and likelihood of stent implantation (*p*=0.003) as well as number of implanted stents (*p*=0.020) were positively correlated (Supplementary Data S1). The stent rate increases from 33.71% for the lowest volume centers with ≤100 interventions compared to 58.59% for the highest volume centers with >1000 interventions. The average number of stents increases in parallel from 0.56 to 1.11. This means that undergoing coronary angiography in a high volume center is associated with an increased likelihood of stent implantation and a higher number of implanted stents.

**Figure 3:**
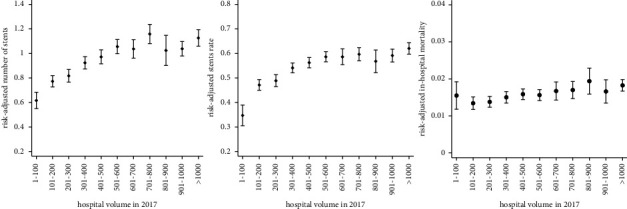
Nonlinear influence of coronary angiography center volume on stent implantation.

However, there was no relationship between center volumes and in-hospital mortality (*p*=0.105), length of hospital stay (*p*=0.201), and reimbursement (*p*=0.108). Major predictors for all endpoints were STEMI and NSTEMI. The analysis of the nonlinear influence of intervention volumes on the likelihood and number of stent implantations suggests a ceiling effect: In hospitals with ≤100 interventions, the likelihood and number of implanted stents per patient are lowest (∼34.4% and ∼0.62, respectively; Figure 3). After that, both rise steadily until a volume of approximately 500 procedures. Finally, the likelihood and the number of stent implantations remain relatively stable in the categories with more than 500 interventions (∼60% and ∼1.07, respectively).

### 3.3. Other Predictors of Stent Implantation

As shown in the full multivariable regression models in the Supplementary Data S1, the most prominent additional predictor of likelihood and number of stent implantations was the presence of STEMI or NSTEMI. Moreover, the likelihood and number of stent implantations are increased for unstable angina pectoris and 2-/3-vessel CAD as well as women and patients of advanced age.

## 4. Discussion

Our study shows mixed results regarding a volume-outcome relationship among patients undergoing coronary angiography in German hospitals. First of all, low volume centers take comparatively more frequent care of emergency cases and less frequent care of complex cases such as 3-vessel or left main disease. This results in lower use of PCI and stents with comparable risk for in-hospital mortality as patients hospitalized in high volume centers and serves as a reminder that these centers potentially fulfil an important purpose by reducing travel times for emergency patients. Secondly, we found that the likelihood and number of implanted stents per patient continuously increase with volume until the threshold of 500 interventions per center and year, consistent with increasing complexity of cases due to a higher proportion of 3-vessel, left main disease, and in-stent stenosis. Therefore, a selection effect can be assumed. Thirdly, clear evidence in favor of mandatory minimum thresholds, usually discussed as >400 procedures per year and center [5], was not identified.

The trend towards a shorter length of stay in high volume centers suggests that patients recover faster after the procedure. This can have a positive impact on their quality of life and could thus be considered a point in favor of high volume centers.

In addition, there was no relationship between center volumes and in-hospital mortality. However, in-hospital mortality was significantly associated with STEMI, NSTEMI, left main stenosis, and emergencies. Accordingly, in-hospital mortality appears to be primarily determined by the severity of the underlying disease.

Recently, a study by Hulme et al. [17] did not find an inverse volume-outcome relationship in operator volume regarding the endpoint of mortality in 133,970 procedures from England and Wales, and O'Neill et al. [18] also found no volume-outcome relationship at the center level regarding the endpoint mortality in the UK healthcare system, which corresponds to our results.

In Japan, Inohara et al. [19] found an inverse volume-outcome relationship, with patients treated in centers of the lowest procedure volume category (<150 PCIs per year) being at increased risk for in-hospital mortality and a composite endpoint including in-hospital death and periprocedural complications in a national Japanese registry. Kodaira et al. [20], in contrast, observed no such association in a multicenter registry from the Tokyo area.

In the US, Badheka et al. [21] found an inverse volume-outcome relationship regarding the endpoints in hospital mortality, periprocedural complications, length of hospital stay, and cost of hospitalization in a cohort of 457,498 procedures performed in 2005–2009. Fanaroff et al. [22] found an inverse volume-outcome relationship in operator volume regarding the endpoint in-hospital mortality but not for the endpoint postdischarge major adverse coronary events which included all-cause death, rehospitalization for myocardial infarction, and unplanned coronary revascularization in a cohort of 723,644 procedures performed between 2009 and 2014. According to our results, more emergency procedures were performed in low volume centers. Qian et al. [23] analyzed the volume-outcome relationship regarding mortality and the appropriateness of PCI in a study of 144,196 patients from 63 nonfederal hospitals in New York between 2012 and 2015. Qian et al. found no significant volume-outcome relationship regarding in-hospital mortality but strong association between hospital volume and rates of inappropriate PCIs. For a threshold of 500 procedures per year and center, for instance, Qian et al. report an inappropriateness rate of 7.52% for patients treated in low volume centers and 10.35% for patients treated in high volume centers (*p* < 0.001).

### 4.1. Limitations

In line with previous analyses [10, 24–26], our study also has some limitations: The basis of our study is administrative data and, therefore, coding errors are nearly unavoidable. The risk adjustment also contained parameters whose completeness cannot be totally guaranteed. For instance, information about individual stent types was not available in the dataset. Based on the data provided by the Federal Bureau of statistics, no local assignment of the analyzed centers and their volumes can be provided. Moreover, we can only compare the center level but not the individual interventionalist level, i.e., the impact of the interventionalist's experience and volume.

## 5. Conclusions

In summary, the presented evidence challenges the recommendation of minimum volume cutoffs at the center level. The present study suggests that (1) smaller, dislocated centers with lower volumes could be seen as equally necessary for emergency cases. The concentration of procedures to high volume centers may result in loss of time and in costly transfers of patients. (2) Higher volume centers treat more complex cases using more stent implantations with stable in-hospital mortality and safety. Beyond that it is not clear how and by whom an optimal threshold might be defined, how the influence of interventionalist or center volumes could be combined, and how new centers could enter the market [10, 27]. Thus, high and low volume centers have their justification. This should be kept in mind when discussing a minimum quantity for coronary angiography.

## Figures and Tables

**Table 1:** Baseline characteristics per center category in 2017.

	Total	≤100	101–200	201–300	301–400	401–500	501–600	601–700	701–800	801–900	901–1000	>1000
Number of patients	528,188	11,049	24,567	46,352	57,755	49,952	60,282	48,986	40,061	37,860	40,090	111,234
Number of centers	828	281	75	90	89	63	63	43	31	25	24	44
Logistic EuroSCORE	8.43	9.28	8.71	8.43	8.53	8.38	8.61	8.46	8.26	8.09	8.68	8.22
Age in years	69.79	70.74	70.03	69.90	69.79	69.60	69.82	69.93	69.69	69.30	69.75	69.83
Female	29.27%	31.25%	31.62%	30.22%	30.19%	28.90%	29.77%	28.43%	29.58%	27.54%	29.32%	28.40%
1‐vessel CAD	26.57%	31.95%	30.30%	28.53%	28.45%	27.35%	26.21%	26.22%	25.62%	25.95%	25.93%	24.22%
2‐vessel CAD	28.33%	28.20%	28.76%	29.31%	28.58%	29.39%	28.65%	28.71%	28.29%	28.57%	27.84%	27.00%
3‐vessel CAD	45.39%	39.52%	40.81%	42.07%	43.17%	43.55%	45.27%	45.66%	46.34%	46.01%	47.23%	49.08%
Left main stenosis	7.42%	5.33%	6.28%	5.67%	6.53%	6.30%	6.73%	8.45%	6.70%	7.71%	8.71%	9.18%
Stable AP	1.10%	1.82%	1.44%	1.96%	1.32%	1.43%	1.35%	0.52%	1.09%	0.95%	0.66%	0.65%
Unstable AP	13.03%	17.68%	16.45%	14.34%	13.70%	14.56%	13.79%	12.26%	12.80%	12.06%	11.11%	11.28%
NSTEMI	20.14%	23.60%	23.52%	21.93%	22.93%	22.05%	22.13%	19.43%	18.20%	19.29%	19.26%	16.52%
STEMI	11.20%	5.50%	10.50%	11.21%	12.69%	13.19%	11.69%	11.38%	10.80%	11.72%	10.45%	10.14%
In‐stent stenosis	4.43%	1.70%	2.65%	3.16%	3.11%	3.57%	3.74%	4.77%	4.21%	4.76%	5.76%	6.42%
Emergency admission	50.12%	53.91%	56.64%	52.46%	54.02%	53.64%	54.28%	49.60%	48.10%	46.02%	47.75%	44.69%

AP: angina pectoris; CAD: coronary artery disease; EuroSCORE: European system for cardiac operative risk evaluation; NSTEMI: non-ST-segment elevation myocardial infarction; STEMI: ST-segment elevation myocardial infarction.

**Table 2:** In-hospital outcomes per center category in 2017.

	Total	≤100	101–200	201–300	301–400	401–500	501–600	601–700	701–800	801–900	901–1000	>1000
Number of patients	528,188	11,049	24,567	46,352	57,755	49,952	60,282	48,986	40,061	37,860	40,090	111,234
Number of stents, mean	1.01	0.56	0.77	0.82	0.95	1.00	1.07	1.03	1.15	1.03	1.02	1.11
Stent rate (at least one stent), %	55.22%	33.71%	47.26%	48.93%	54.19%	56.39%	57.60%	56.58%	57.41%	55.40%	56.32%	58.59%
In‐hospital mortality, %	2.93%	2.41%	2.47%	2.49%	2.88%	3.02%	2.93%	3.04%	2.94%	3.30%	2.93%	3.06%
Length of hospital stay, mean	6.46	7.88	6.43	6.23	6.42	6.44	6.30	6.60	6.63	7.30	6.23	6.22
Reimbursement, mean	5,531 €	4,760 €	4,404 €	4,477 €	4,907 €	5,197 €	5,069 €	5,674 €	5,677 €	6,820 €	5,795 €	6,378 €

## Data Availability

The data used in this study are available upon reasonable request from the corresponding author. The patients' data are stored in the server of the Federal Bureau of statistics and are not available due to data protection. The calculated raw data will be sent anonymized to the scientists.

## Abbreviations

AP: Angina pectoris
CAD: Coronary artery disease
DESTATIS: Research Data Center of the Federal Bureau of Statistics
DRG: Diagnosis-related groups
EuroSCORE: European system for cardiac operative risk evaluation
NSTEMI: Non-ST-segment elevation myocardial infarction
OPS: German operation and procedure classification
PCI: Percutaneous coronary intervention
STEMI: ST-segment elevation myocardial infarction.

## Supplementary Materials

Supplementary Materials S1: multiple linear or logistic regression analyses and nonlinear relationship of center volumes to all observed outcomes.
