# Supplementary material for: Center Volumes Correlate with Likelihood of Stent Implantation in German Coronary Angiography
Source: J Interv Cardiol. 2023 Nov 9;2023:3723657. doi: 10.1155/2023/3723657 (PMC10653957; doi:10.1155/2023/3723657)

Center volumes correlate with likelihood of stent implantation in German coronary angiography

Vera Oettinger, MD, M.Sc.; Philip Hehn; Christoph Bode, MD; Manfred Zehender, MD, PhD; Constantin von zur Mühlen, MD; Dirk Westermann, MD; Peter Stachon, MD; Klaus Kaier, PhD

Supplementary Data S1: Multiple linear or logistic regression analyses and non-linear relationship of center volumes to all observed outcomes

Multiple linear or logistic regression analyses

	Number of Stents				Stent rate (at least one stent)				In-hospital mortality				Length of stay				Reimbursement			
	Coeff	p-value	95%CI		OR	p-value	95%CI		OR	p-value	95%CI		Coeff	p-value	95%CI		Coeff	p-value	95%CI	
**Hospital volume (linear relationship per 100 procedures)**	**0.01**	**0.020**	**0.00**	**0.02**	**1.02**	**0.003**	**1.01**	**1.03**	**1.01**	**0.105**	**1.00**	**1.01**	**-0.01**	**0.201**	**-0.03**	**0.01**	**58 €**	**0.108**	**-13 €**	**128 €**
**Logistic EuroSCORE**	-0.01	0.000	-0.02	-0.01	0.97	0.000	0.97	0.97	1.02	0.000	1.02	1.02	0.24	0.000	0.23	0.25	151 €	0.000	134 €	167 €
**Age in 10 years**	0.03	0.000	0.02	0.03	1.03	0.000	1.01	1.04	1.40	0.000	1.37	1.44	-0.08	0.002	-0.13	-0.03	-181 €	0.000	-25 €	-12 €
**Female**	0.02	0.000	0.01	0.03	1.05	0.000	1.03	1.07	0.96	0.034	0.92	1.00	-0.19	0.000	-0.25	-0.12	-544 €	0.000	-6,181 €	-4,699 €
**1-vessel CAD**	-0.23	0.000	-0.28	-0.18	0.80	0.000	0.73	0.88	0.65	0.000	0.52	0.82	3.02	0.000	2.50	3.54	2,820 €	0.000	2,149 €	3,490 €
**2-vessel CAD**	0.10	0.000	0.05	0.15	1.28	0.000	1.16	1.41	0.73	0.005	0.59	0.91	2.90	0.000	2.38	3.42	2,873 €	0.000	2,206 €	3,540 €
**3-vessel CAD**	0.19	0.000	0.14	0.24	1.08	0.112	0.98	1.19	1.07	0.547	0.86	1.33	3.20	0.000	2.68	3.73	3,206 €	0.000	2,529 €	3,883 €
**Left main stenosis**	-0.11	0.000	-0.16	-0.07	0.59	0.000	0.55	0.63	1.67	0.000	1.57	1.77	1.49	0.000	1.24	1.74	1,609 €	0.000	1,265 €	1,954 €
**Stable AP**	-0.04	0.216	-0.11	0.02	0.96	0.494	0.84	1.09	0.29	0.000	0.20	0.42	-1.01	0.000	-1.33	-0.69	-1,238 €	0.000	-1,512 €	-965 €
**Unstable AP**	0.16	0.000	0.13	0.19	1.54	0.000	1.46	1.63	0.39	0.000	0.35	0.43	-1.10	0.000	-1.23	-0.96	-1,137 €	0.000	-1,276 €	-998 €
**NSTEMI**	0.54	0.000	0.52	0.56	3.07	0.000	2.95	3.20	2.60	0.000	2.44	2.77	1.93	0.000	1.79	2.07	2,279 €	0.000	2,144 €	2,414 €
**STEMI**	0.98	0.000	0.95	1.00	10.01	0.000	9.52	10.54	8.47	0.000	7.93	9.04	2.01	0.000	1.86	2.17	3,183 €	0.000	3,006 €	3,361 €
**In-stent stenosis**	-0.03	0.090	-0.06	0.00	1.06	0.059	1.00	1.12	0.88	0.007	0.81	0.97	-0.60	0.000	-0.75	-0.45	-346 €	0.000	-524 €	-168 €
**Emergency admission**	-0.03	0.007	-0.06	-0.01	0.91	0.000	0.87	0.95	1.43	0.000	1.33	1.54	1.04	0.000	0.84	1.23	-305 €	0.015	-552 €	-58 €

Non-linear relationship

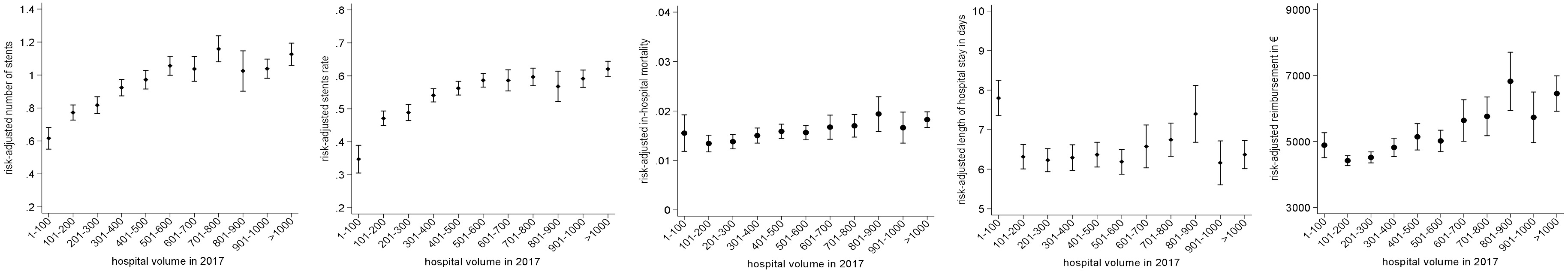

Supplement: Supplementary Materials — S1: multiple linear or logistic regression analyses and nonlinear relationship of center volumes to all observed outcomes. [file 3723657.f1.pdf]
